# COMBINING TRANSCRANIAL DIRECT CURRENT STIMULATION AND ROBOTIC-ASSISTED TRAINING TO ADDRESS UPPER EXTREMITY DEFICITS IN ACUTE DISSEMINATED ENCEPHALOMYELITIS: A CASE REPORT

**DOI:** 10.2340/jrm-cc.v8.42152

**Published:** 2025-01-21

**Authors:** Maureen AHIATSI, Matthieu VINCENOT, Christian BOCTI, Guillaume LÉONARD, Marie-Hélène MILOT

**Affiliations:** 1 Research Centre on Aging, Centre intégré universitaire de santé et de services sociaux de l’Estrie – Centre hospitalier universitaire de Sherbrooke (CIUSSS de l’Estrie – CHUS), Sherbrooke, Québec, Canada J1H 4C4; 2 School of Rehabilitation, Université de Sherbrooke, Sherbrooke, Québec, Canada J1H 5N4; 3 Division of Neurology, Department of Medicine; 4 Faculty of Medicine and Health Sciences, Université de Sherbrooke, Sherbrooke, Québec, Canada J1H 5N4

**Keywords:** acute disseminated encephalomyelitis, case report, motor function, transcranial direct current stimulation, robotic-assisted training, upper limb

## Abstract

A 45-year-old woman with persistent acute disseminated encephalomyelitis sequelae participated in a 4-week robotic-assisted training program of her affected arm combined with transcranial direct current stimulation. Clinical indicators such as range of motion, motor function of the affected arm, fatigue, pain, spasticity, and quality of life were assessed pre/post-intervention. The results demonstrated clinical benefits post- intervention, with an improvement in range of motion and affected arm motor function, fatigue, and quality of life of the patient. Although preliminary, the results of this case report support the development of innovative technologically assisted rehabilitative strategies for individuals with acute disseminated encephalomyelitis sequelae, including a robot-assisted rehabilitation program coupled with neurostimulation sessions. Further large-scale randomized controlled trials are needed to confirm these findings and rigorously assess the efficacy of this approach in acute disseminated encephalomyelitis individuals.

## LAY ABSTRACT

A 45-year-old woman had residual motor deficits after a rare disease called acute disseminated encephalomyelitis. Despite 4 years of rehabilitation, her condition did not improve. She tried an intervention combining robotic-assisted training program of her affected arm with non-invasive brain stimulation called transcranial direct current stimulation. Before and after the intervention, her arm movement, fatigue, pain, and quality of life were assessed. After the intervention, she showed improvements, including better arm movement and less fatigue. Although this is a case report, the results are promising and suggest that this combined intervention could be a new way in clinical practice to help people with long-term effects from acute disseminated encephalomyelitis. However, further studies are needed to confirm these findings.

Acute disseminated encephalomyelitis (ADEM) is an inflammatory demyelinating disease, affecting the central nervous system (1), specifically damaging the myelin sheath – the specialized membrane of glial cells (oligodendrocytes) around axons – whose role is to isolate the axon to facilitate and accelerate action potential conduction. This monophasic autoimmune pathology is usually triggered by viral or bacterial infections or after vaccination. Although more common in pediatric population, ADEM can also be observed in adults (1). The incidence of ADEM is between 0.07 and 0.64 cases per 100,000 children annually (1).

Acute disseminated encephalomyelitis onset is abrupt and combines encephalopathy signs with neurological symptoms of progressive evolution (2). Diagnosis is mainly exclusionary, based on brain imaging, biological markers and clinical examination (1). Cerebral imaging typically shows widespread white matter lesions in *FLAIR* sequences, while cerebrospinal fluid analysis may uncover an inflammatory profile (increase in white blood cells or proteins). Clinical examination can reveal encephalitic signs (1) like disturbed consciousness, convulsion and fever, associated with focal neurological impairments (hemiplegia, pyramidal syndrome and visual deficits). ADEM symptoms vary in intensity, but can progress rapidly, requiring hospitalization in an intensive care unit in the most severe cases.

There is no standard treatment for ADEM (1). The current approach is mainly pharmacological, involving intravenous corticosteroids, immunoglobulins, and plasma exchange. Prognosis is generally favorable, but less so in adults (1), who often experience residual focal motor deficits similar to those observed in multiple sclerosis (MS). Physical therapy and exercises are crucial in the recovery of severely affected ADEM individuals, for motor function and coordination (3). Although rehabilitation and exercises are essential, chronic residual deficits persist. Combining rehabilitation with neurostimulation techniques, such as transcranial direct current stimulation (tDCS), has proven effective in enhancing functional improvements across various neurological populations (4), and may be therefore a promising approach to augment the benefits of rehabilitation exercises in ADEM.

tDCS is a non-invasive neuromodulation technique that modulates cortical excitability via an excitatory anode and an inhibitory cathode using low direct current (1–2 mA). Today, it is among the most widely studied technique because of its non-invasive nature, affordability, ease of integration with other therapies, and potential positive long-term effects. For MS patients, tDCS combined with physical rehabilitation has improved motor recovery, reduced fatigue (5) and enhanced quality of life (6). In addition, the integration of innovative rehabilitation technologies, such as robot-assisted therapies (RAT), has demonstrated significant benefits, providing precise movement and force application to the affected limb, often paired with visual feedback, allowing patients to perform repetitive exercises in an engaging and interactive manner. Previous studies have demonstrated the effectiveness of RAT in improving motor function, walking and physical function (7) in populations with motor impairments (e.g. stroke, MS). Knowing that RAT can promote motor recovery through targeted training, while tDCS can enhance neuronal plasticity through neuromodulation, the combination of tDCS and RAT represents an innovative approach to treating ADEM pathology by acting on neuroplasticity. To date, however, the effects of tDCS and RAT in ADEM individuals remain undocumented.

Here, we describe the case of a 45-year-old woman diagnosed with ADEM, who presented residual motor deficits several years after traditional rehabilitation care and in whom positive effects could be observed following a rehabilitation approach combining tDCS with a robot-assisted training program.

## CASE PRESENTATION

The patient, a 45-year-old university administrative assistant with no past medical history (except iron deficiency anemia diagnosed in 2012 with a negative workup) and no regular medication intake. She had no family history of neurological disease.

On 29^th^ December 2014, she went to the emergency department, complaining of paraesthesia that began 5 days earlier in her right upper limb (UL) and rapidly spread to her entire right hemibody. She also reported decreased muscle strength (paresis) with a course similar to that of her paraesthesia and ataxia while walking. She was quickly referred to the hospital’s neurology department. An initial brain MRI on 30^th^ December showed central pons FLAIR hypersignal with diffusion restriction. Initial differential diagnosis included demyelination, ischemia, or infectious rhombencephalitis. Extensive cerebrospinal fluid analysis for infectious agents and aquaporin-4-specific immunoglobulin both returned negative results and anti-MOG antibodies also. Treatments with intravenous hydrocortisone (100 mg/day for 5 days), immunoglobulins and cyclophosphamides were started the following day.

Despite treatment, the patient’s condition worsened within the first week, with increased right hemiparesis, left-sided ataxia, dysarthria and dysphagia. A follow-up brain MRI performed 1 week after admission with gadolinium enhancement showed more diffuse white matter lesions, compatible with ADEM (see Table I). The initial clinical picture and the evolution of the clinical manifestations led to the diagnosis of ADEM by the neurologist. Over the next 2 weeks, dysphagia and dysarthria rapidly worsened necessitating a nasogastric tube for safe feeding. Right hemiparesis also worsened. Due to her condition’s severity, she was transferred to the intensive care unit for a close monitoring, though intubation was not required. After 2 days, intravenous corticosteroids were resumed and plasmapheresis sessions were added. She underwent 3 months of rehabilitation in the neurology department, including physiotherapy, occupational and speech therapy, then received intensive inpatient rehabilitation at a regional rehabilitation centre until 7^th^ August 2015, followed by 10 hours/week of outpatient rehabilitation (physiotherapy, occupational therapy and psychological support) until January 2016, when recovery plateaued.

**Table I:** Clinical evolution observed through MRI scans performed during hospitalization

MRI dates	Clinical description
30^th^ December 2014	Central pons FLAIR hypersignal with diffusion restriction
8^th^ January 2015	Extension of the size of the pontine lesion occupying most of the pons with extension within the cerebellar peduncles; evidence of microhaemorrhages within the lesion; persistence of diffusion restriction and peripheral gadolinium enhancement.
8^th^ January 2015	Negative spinal MRI
26^th^ January 2015	Progression of the lesions to the cerebral peduncles; increase in size of lesions in the cerebellar peduncles, with caudal extension toward the medulla; mild mass effect compatible with edema around the lesion (see Fig. 1)
4^th^ February and 19^th^ February 2015	Improvement and reduction of gadolinium enhancement; reduction of the size of diffusion-restricted lesion
6^th^ July 2016	Atrophy and persistence of diffuse abnormal FLAIR signal of the pons and middle cerebellar peduncles, compatible with chronic phase of demyelinating event; no evidence of new lesion

MRI: magnetic resonance imaging.

**Fig. 1:**
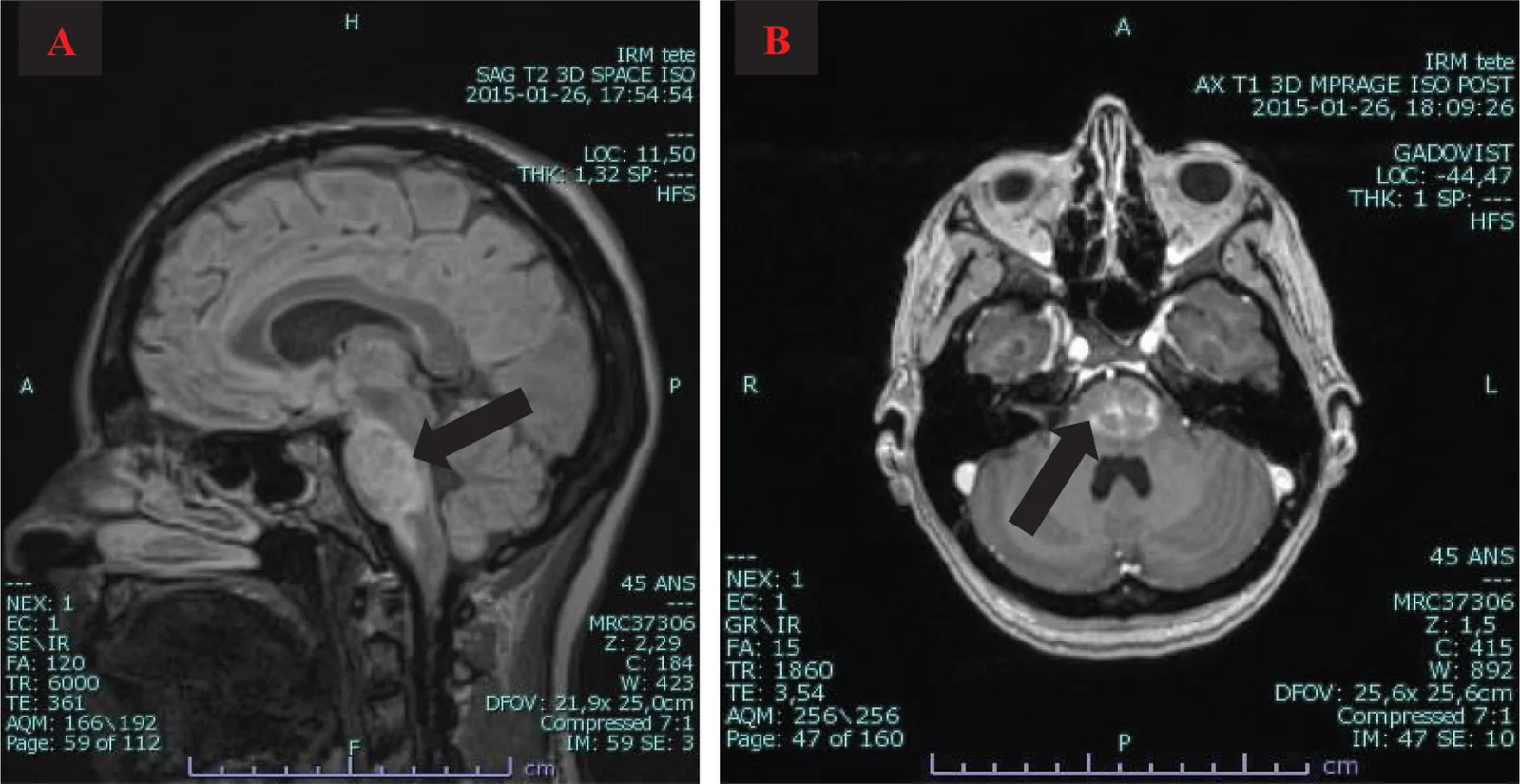
Magnetic resonance imaging (MRI) findings. (A) Sagittal MRI showing FLAIR hypersignal in pons and medulla; (B) Axial T1 MRI with gadolinium showing enhancement of the pontine lesion.

The MRI from 16^th^ July 2016 revealed significant pontine atrophy and persistent FLAIR signal abnormality in the pontine and cerebellar peduncles. The neurologist provided annual outpatient neurological follow-ups (1–2 per year), with no new recurrence of inflammatory episodes by her last visit in March 2022, confirming the same ADEM diagnosis since her discharge in 2015. Four years post-onset, a slow improvement in motor function was observed, including a reduction in dysarthria and a slight increase in her ability to initiate leg swing, with no change in spasticity and fasciculations. Given her persisting deficits and high motivation, the possibility of resuming rehabilitation with innovative approaches such as tDCS combined with physical rehabilitation was discussed. She was referred to the Research Centre on Aging (Sherbrooke, Quebec, Canada), where a specialized research team in neurorehabilitation and neurostimulation designed a training protocol for her involving tDCS coupled with a robotic arm training of her most affected arm (8).

### Intervention

The intervention occurred from October to November 2022 at the Research Centre on Aging. Pre/post-intervention, the patient completed clinical assessments, including tests on her right arm, fatigue level and quality of life (see Table II). Written informed consent was obtained during her first visit, and all procedures followed the Declaration of Helsinki and the Tri-Council Policy Statement – Ethical Conduct for Research Involving Humans (TCPS 2). Ethics approval was granted by the CIUSSS de l’Estrie-CHUS Research Ethics Committee.

**Table II:** Measurement tools, their outcomes and metrics for collecting patient data

Tool	Outcome	Metrics
Goniometer	Range of motion of affected arm: Active Passive	Degree Movements assessed: Shoulder flexion Elbow flexion Wrist extension
Pain Visual Analog Scale	Pain intensity	0–10 scale (0 = no pain and 10 = worst possible pain)
Modified Ashworth Scale (MAS)	Spasticity	5 levels ranging from 0 (no increase in muscle tone) to 4 (segment is rigid in flexion or extension) Muscle groups assessed: shoulder, elbow, wrist, finger and thumb flexors
Amended Motor Club Assessment (AMCA)	Arm motor function and performance	16 items for upper limb activities rated on a 3-point scale from 0 (no movement) to 2 (full range of movement compared to the extremity of the other side of the body)
Short Form 36 (SF-36)	Quality of life	Multi-level Likert scale measuring 8 components: physical functioning, physical role, body pain, general health, vitality, social functioning, emotional role and mental health
Modified Fatigue Impact Scale (MFIS)	Impact of the level of fatigue on the patient’s daily activities	21 questions using 5-level scale ranging from 0 (never) to 4 (always)

MAS: Modified Ashworth Scale; AMCA: Amended Motor Club Assessment; SF-36: Short Form 36; MFIS: Modified Fatigue Impact Scale.

The 4-week training program (3 times/week; 60 min/session) focused on the patient’s right UL, using a robotic arm named *Exerciser for Rehabilitation of the Arm* (ERA) (8), coupled with tDCS. The training program followed MS exercise recommendations and was administered by a trained therapist.

**Fig. 2:**
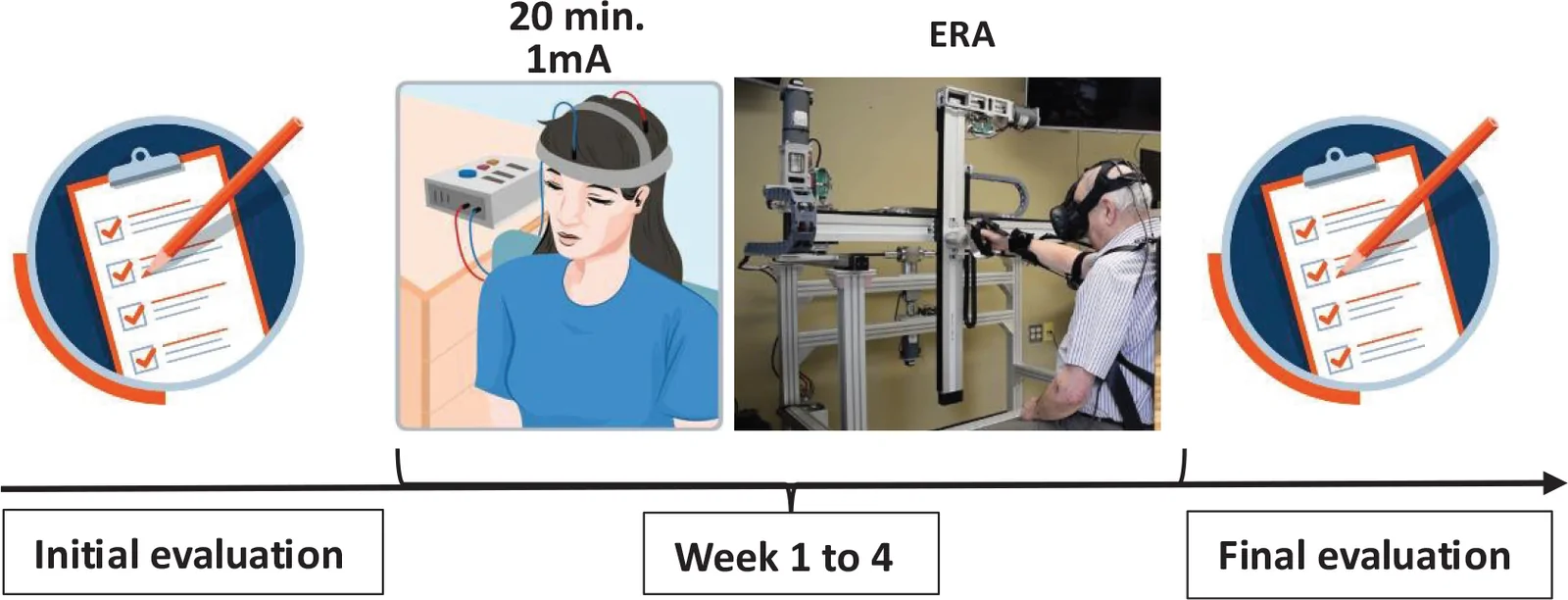
Project timeline. Initial evaluation of the patient (arm function and performance, fatigue level and quality of life). Week 1 to 4 consisting of the robotic ERA arm training (60 min per session, 3X/week, 4 weeks) combined to tDCS . Fiinal evaluation of the patient (arm function and performance, fatigue level and quality of life). ERA: exerciser for rehabilitation of the arm; Min: minute; mA: milli-ampere

ERA, an end-effector robot, enables 3D assisted reaching movements in a virtual environment using a virtual reality headset (HTC Vive: DR globalTech Inc.). The patient’s arm was supported in an armrest (Kinova Robotics, Canada) and her hand was secured with Velcro to the ERA’s handle. Her trunk was stabilized with straps to ensure proper stabilization and positioning during training (see Fig. 2). The training program included 2 phases: a calibration phase to adapt ERA workspace to the patient’s range of motion abilities, followed by a training phase where she aimed at targets appearing randomly at different locations in the virtual environment. The goal was to reach the targets as quickly and accurately as possible (see Fig. 2), with ERA providing assistance-as-needed and allowing the patient to rest at any time without moving her arm away from the target.

Before each training session, tDCS (*Soterix Medical*, New York, USA) was applied to the patient’s scalp in an anodal montage for 20 min (1 mA, 12 sessions). To do this, 5 × 7 cm saline-soaked anode (35 mm²) and cathode electrodes (35 mm²) were placed on the ipsilesional motor cortex (left in the case of our patient) and contralateral supraorbital region, respectively.

## RESULTS

Following the robotic training and tDCS intervention, the patient demonstrated improvements in her passive range of motion and motor function (in a lying down position) of her affected arm, along with a reduced fatigue level and enhanced quality of life. Additionally, her performance in the robotic-assisted workspace environment, in which she performed her training, improved, though a slight increase in spasticity in her wrist and fingers was noticed (see Table III).

**Table III:** Clinical variables changes pre- and post-tDCS combined with robotic arm training of the affected upper limb

Clinical outcomes	Pre	Post
**Modified Ashworth Scale (MAS) (/4)**		
Shoulder extensors (normal = 0)	1	1
Elbow flexors (normal = 0)	1	1
Wrist flexors (normal = 0)	0	1
Fingers flexors (normal = 0)	0	1
**Range of motion of affected arm (°)**		
Shoulder flexion		
Active	0	0
Passive	110	**135**
Elbow flexion		
Active	100	**118**
Passive	130	NR
Wrist flexion		
Active	0	0
Passive	55	**65**
**Amended Motor Club Assessment (AMCA)**		
Left arm		
Lying down position (/6)	6	6
Seated position (/20)	20	20
Right arm (affected arm)		
Lying position (/6)	2	**4**
Seated position (/20)	4	4
**Modified Fatigue Impact Scale (MFIS)**		
Physical subscale (/36)	21	**0**
Cognitive subscale (/40)	8	**0**
Psychosocial subscale (/8)	4	**0**
MFIS total score (/84)	33	**0**
**Pain assessment (/10)**	0	0
**Short Form 36 (SF36)**		
Physical functioning (/100)	15	**35**
Role limitations due to physical health (/100)	100	100
Role limitation due to emotional problems (/100)	100	100
Energy/Fatigue (/100)	65	**80**
Emotional well-being (/100)	88	**92**
Social functioning (/100)	100	100
Pain (/100)	100	100
General health (/100)	85	85
**Amplitude of workspace customized to the patient’s abilities†**		
X axis (cm)	9	**23**
Y axis (cm)	27	**55**
Z axis (cm)	22	**26**

Numbers in bold depict improvement.

NR: the patient’s elbow could not be moved to the required starting position, making accurate measurement impossible; AMCA: Amended Motor Club Assessment; MAS: Modified Ashworth Scale; MFIS: Modified Fatigue Impact Scale; SF-36: Short Form 36.

† The amplitude of workspace was determined during the calibration phase. During this phase, the patient was asked to move the robot’s handle to enlarge as much as possible a 3D cube seen in the headset. The final size of the cube represented the available range of motion of the patient that the robot then used for training.

## DISCUSSION

Our case report illustrates improvements in motor function, fatigue level and quality of life in an adult ADEM patient following a 4-week robotic training program coupled with 12 tDCS sessions. Unfortunately, few studies have documented the ADEM management in adults, particularly in the field of rehabilitation, making it difficult to compare our observations with other studies.

Post-intervention, motor function in the affected arm improved, with increased passive range of motion and improved movement of the affected arm while lying down, despite a slight spasticity increase. This observed increase in spasticity may be due to various factors, such as posture during the assessment, or the patient’s mental state (9). These gains improved performance in the robotic-assisted workspace environment and significantly reduced fatigue level. These significant improvements contrast with the patient’s poor progress over the previous 4 years, despite a combination of pharmacological treatments and rehabilitation. Given the importance of highly repetitive and intense training to promote recovery in chronic CNS diseases (4, 6), repetition and intensity may also be crucial for promoting recovery in chronic ADEM patients.

These observations are somewhat reminiscent of the results of studies showing positive effects of RAT on recovery of individuals with other neurological conditions such as MS (7). A meta-analysis by Xie et al. examined whether patients with MS benefited more from robot-assisted gait training compared to conventional gait therapy (7). Their results revealed that robot-assisted gait training provided greater walking improvements for these patients. Regarding tDCS, it has proven to be effective in improving motor function, such as gait ability, and reducing fatigue in MS population (5, 10). However, some studies have reported less convincing results from tDCS, suggesting limited or non-significant benefits in improving recovery (4).

The positive effects seen in our patient following tDCS and training is promising for individuals with ADEM sequelae, especially adults who tend to respond less favorably to traditional treatments. Exploring alternative therapeutic approaches for this population is therefore essential, and the positive results of this case report suggest that tDCS and training could be an interesting avenue for improving physical rehabilitation for this population.

Although the results are encouraging, they must be interpreted cautiously. The proposed protocol only assessed immediate changes, leaving long-term effect unexplored. Also, our results could be influenced by other factors, such as the placebo effect, given the patient’s high motivation and expectations (11), which could have influenced how the patient scored on subjective scales such as the MFIS and SF-36 or the effect of care from close supervision and support of the research team (12). Also, our protocol did not have several baseline assessments, which means that we cannot completely rule out the possibility of spontaneous recovery and its impact on the data post-intervention. Nonetheless, our study’s internal validity is supported by a rigorous methodology, with a well-established training and tDCS protocol tested in previous studies. Future studies are needed to validate the current observations and allow improvement of rehabilitation care for ADEM patients.

In conclusion, this case report highlights the possible benefits of a multimodal approach (training and tDCS) for managing impairments in an adult with ADEM. It also highlights the need for further research to evaluate the efficacy and long-term effects of such proposed intervention. Future studies would therefore be needed to confirm the results observed and optimize the intervention protocol for individuals with ADEM.
